# XIAP BIR domain suppresses miR-200a expression and subsequently promotes EGFR protein translation and anchorage-independent growth of bladder cancer cell

**DOI:** 10.1186/s13045-016-0376-9

**Published:** 2017-01-05

**Authors:** Chao Huang, Xingruo Zeng, Guosong Jiang, Xin Liao, Claire Liu, Jingxia Li, Honglei Jin, Junlan Zhu, Hong Sun, Xue-Ru Wu, Chuanshu Huang

**Affiliations:** 1 Nelson Institute of Environmental Medicine, New York University School of Medicine, 57 Old Forge Road, Tuxedo, NY 10987 USA; 2 Zhejiang Provincial Key Laboratory for Technology & Application of Model Organisms, School of Life Sciences, Wenzhou Medical University, Wenzhou, Zhejiang China 325035; 3 Departments of Urology and Pathology, New York University School of Medicine, New York, NY 10016 USA; 4 VA Medical Center in Manhattan, New York, NY 10010 USA

**Keywords:** XIAP BIR domain, miRNA, EGFR, Rac1, Bladder cancer

## Abstract

**Background:**

The X-linked inhibitor of apoptosis protein (XIAP) is a well-known potent apoptosis suppressor and also participates in cancer cell biological behaviors, therefore attracting great attentions as a potential antineoplastic therapeutic target for past years. Anti-IAP therapy is reported to be closely related to epidermal growth factor receptor (EGFR) expression level. However, whether and how XIAP modulates EGFR expression remains largely unknown.

**Methods:**

Human XIAP was knockdown with short-hairpin RNA in two different bladder cancer cell lines, T24T and UMUC3. Two XIAP mutants, XIAP ∆BIR (deletion of N-terminal three BIR domains) and XIAP ∆RING (deletion of C-terminal RING domain and keeping the function of BIR domains), were generated to determine which domain is involved in regulating EGFR.

**Results:**

We found here that lacking of XIAP expression resulted in a remarkable suppression of EGFR expression, consequently leading to the deficiency of anchorage-independent cell growth. Further study demonstrated that BIR domain of XIAP was crucial for regulating the EGFR translation by suppressing the transcription and expression of miR-200a. Mechanistic studies indicated that BIR domain activated the protein phosphatase 2 (PP2A) activity by decreasing the phosphorylation of PP2A at Tyr307 in its catalytic subunit, PP2A-C. Such activated PP2A prevented the deviant phosphorylation and activation of MAPK kinases/MAPKs, their downstream effector c-Jun, and in turn inhibiting transcription of c-Jun-regulated the miR-200a.

**Conclusions:**

Our study uncovered a novel function of BIR domain of XIAP in regulating the EGFR translation, providing significant insight into the understanding of the XIAP overexpression in the cancer development and progression, further offering a new theoretical support for using XIAP BIR domain and EGFR as targets for cancer therapy.

## Background

The X-linked inhibitor of apoptosis protein (XIAP) is a member of the inhibitors of apoptosis (IAP) family, which mainly function as suppressors of apoptosis. XIAP is aberrantly increased in a variety of human cancers, including acute leukemia [1], ovarian carcinoma [2], bladder cancer [3], clear cell renal cancer [4, 5], and many other cancers [6–8]. Elevated expression of XIAP mediates the resistance of cancer cells to chemotherapeutic drugs, as well as radiotherapy [9, 10]. Downregulation of XIAP has been shown to sensitize drug-resistant cancer cells to chemotherapeutic agents-induced apoptosis [11–13]. Therefore, as an important diagnostic and prognostic biomarker in many cancers [3, 14], XIAP may serve as a potential therapeutic target for antineoplastic therapy.

XIAP contains three baculovirus IAP repeat domains (BIR 1-3) in the N-terminal of the protein and one RING domain in the C-terminal. In which, BIR1 interacts with proteins that modulate NF-kappaB signaling [15] and BIR2 and BIR3 are critical for the interaction with either caspase-3 or -7 (BIR2) or caspase-9 (BIR3), respectively. The RING domain can function as an E3 ligase and mediate the proteasomal degradation of itself and bind to proteins such as caspase-3 or the mitochondrial XIAP-inhibitor SMAC/Diablo [16, 17]. Other than inhibiting apoptosis, XIAP is involved in many cellular functions related to the cancer malignancy. Our recent studies show that XIAP upregulates cyclin D1 via its C-terminal RING domain to promote bladder cancer cell growth [18] and enhances colorectal cancer cell motilities through inhibiting the RhoGDIα SUMOlation at the lys-138 site [19, 20]. Moreover, our recent study reveals that XIAP directly binds to E2F1 via N-terminal BIR domains and enhances E2F1 transcriptional activity, which subsequently promotes colorectal cancer cell growth [21]. In addition, inhibition of XIAP activity by Embelin in bladder cancer cells not only reduces cell viability but also affects cell invasion in vitro, suggesting an important role of XIAP in tumor formation and progression [22].

The epidermal growth factor receptor (EGFR) is a protein mainly existing in the cell membrane and can be activated by binding of its specific ligands, including epidermal growth factor (EGF) and transforming growth factor α (TGFα). EGFR overexpression has been widely reported in a number of cancers, including breast, ovarian, bladder, non-small-cell lung (NSCLC), colorectal cancers, and other tumors [23–29]. Aberrant activation or expression of EGFR leads to the promotion of proliferation, cell motility, and invasive capacity of tumor [30–33]. As a survival signaling protein, EGFR is widely used as a therapeutic target. Many anti-EGFR-based therapeutic drugs have been developed, such as monoclonal antibodies cetuximab and panitumumab and tyrosine kinase inhibitors like gefitinib and afatinib [34], which exhibit the improved therapeutic effect in the patients.

A recent study shows that tumor cells overexpressing EGFR are more sensitive to anti-IAPs therapy [35], suggesting a potential relationship between XIAP and EGFR in cancer cells. However, whether and how XIAP modulates EGFR expression remains largely unknown. In the current study, we found that XIAP BIR domain could regulate EGFR expression through downregulating miR-200a by targeting protein phosphatase 2 (PP2A)/c-Jun axis and further promoted the bladder cancer cell anchorage-independent growth.

## Methods

### Cell culture and reagents

UMUC3 cells and 293 T cells were cultured in DMEM medium (Invitrogen, Carlsbad, CA, USA) supplemented with 10% FBS, and T24T cells were cultured in RPIM1640/F12 medium (Invitrogen, Carlsbad, CA, USA) with 5% FBS. All cells were maintained in a humidified incubator at 37 °C, with a 5% CO2 atmosphere. XIAP polyclonal antibody was purchased from BD PharMingen (San Diego, CA, USA); Antibodies specific against EGFR, STAT3, p52, p65, p50, RelB, c-Jun, p38, Erk1/2, MKK, MEK1/2, and PP2A were bought from Cell Signaling Technology Inc. (Beverly, MA, USA); The specific antibodies against Sp1, E2F1, β-actin and α-Tubulin were from Santa Cruz Biotechnology (Santa Cruz, CA, USA); the antibodies for GAPDH and JNK1/2 were bought from GeneTex, Inc. (Irvine, CA, USA). Okadaic acid was purchased from Santa Cruz Biotechnology (Santa Cruz, CA, USA).

### Plasmids and stable cell transfection

The short-hairpin RNA (shRNA)-specific targeting human XIAP was purchased from Open Biosystems (Lafayette, CO, USA). The overexpression of miR-200a/200b/429 expression plasmid was bought from Addgene (Cambridge, MA, USA). The miR-200a knockdown plasmid was purchased from GeneCopoeia (Rockville, MD, USA). The overexpression of HA-∆RING plasmid, the TAM67 plasmid, a well-characterized dominant-negative c-Jun mutant, and the p100 overexpression plasmid were described in our previously studies [19, 36, 37]. Wild-type EGFR 3′-UTR luciferase reporter is a kind gift from Professor Benjamin Purow (University of Virginia Health System, VA) [38]. The miR-200a binding site point mutation of EGFR 3′-UTR luciferase reporter was constructed in our lab based on the wild-type EGFR 3′-UTR luciferase reporter.

Cell transfections were performed with PolyJet™ DNA In Vitro Transfection Reagent (SignaGen Laboratories, Rockville, MD, USA) according to the manufacturer’s instructions. For stable transfection, cell cultures were subjected to hygromycin B, G418, or puromycin selection according to the resistance of plasmids, and cells surviving were pooled as stable mass transfectants. The miR200a inhibitor lentivirus was packaged in 293T cells with pCMV delta R8.2 and pMD2.G as described in the previous publication [39].

### Anchorage-independent growth assay

Anchorage-independent growth ability was evaluated in soft agar as described in our previous studies [40]. Briefly, 3 ml of 0.5% agar in basal modified Eagle’s medium supplemented with 10% FBS was layered onto each well of 6-well tissue culture plates; 1 ml of 0.35% agar medium with cells (1 × 10^4^ cells) was added to each well on top of the concretionary 0.5% agar layer. Plates were incubated at 37 °C in 5% CO2 for 2–3 weeks, and the colonies with more than 32 cells were scored and are presented as colonies/10^4^ cells.

### Western blot analysis

Whole cell extracts were prepared with the cell lysis buffer (10 mM Tris-HCl, pH 7.4, 1% SDS, and 1 mM Na_3_VO_4_) as described in our previous studies [41]; 50 μg of proteins were resolved by SDS-PAGE, transferred to a PVDF membrane, and probed with the indicated primary antibodies together with the AP-conjugated secondary antibody. Signals were detected by the enhanced chemifluorescence Western blotting system as described in the previous report [42]. The images were acquired by scanning with the phosphorimager (Typhoon FLA 7000 imager; Pittsburgh, PA, USA).

### Luciferase reporter assay

EGFR mRNA 3′ UTR luciferase reporter, miR-200a/200b/429 promoter luciferase reporter, was stably transfected into cultured cells. Luciferase activity was determined by using the luciferase Assay System kit (Promega, Madison, WI, USA). The results were normalized by internal TK signal. All experiments were done in triplicates and the results expressed as mean ± standard error (SE).

### Quantitative real-time PCR

For mRNA detection: total RNA was extracted using the TRIzol reagent as described in the manufacturer’s instructions (Invitrogen, Grand Island, NY, USA); 5 μg total RNA was used for first-strand cDNA synthesis with oligdT primer by SuperScript IV First-Strand Synthesis system (Invitrogen, Grand Island, NY, USA). The PCR was done using PowerUp SYBR Green Master Mix (Invitrogen, Grand Island, NY, USA) specifically. The primers used in this study were *human egfr*, forward: 5′-CCA AGG CAC GAG TAA CAA GC-3′, reverse: 5′-AGG GCA ATG AGG ACA TAA CCA G-3′; and *human gapdh*, forward: 5′-AGA AGG CTG GGG CTC ATT TG-3′, reverse: 5′-AGG GGC CAT CCA CAG TCT TC-3′. The initial activation was performed at 50 °C for 2 min, 95 °C for 10 min, and followed by 40 cycles (95 °C for 15 s, 60 °C for 1 min).

For miRNA detection: cells used for total RNA extraction using miRNeasy Mini Kit (QIAGEN, Valencia, CA, USA); 1 μg total RNA was used for reverse transcription. Analysis of miRNAs was done using miScript PCR system (QIAGEN, Valencia, CA, USA) by QuantStudio Real-time PCR system (Applied Biosystems). The primers were purchased from Invitrogen (Grand Island, NY, USA), and U6 was used for inner control. The initial activation was performed at 95 °C for 15 min and followed by 40 cycles, denaturation at 95 °C for 15 s, annealing at 55 °C for 30s, and extension at 70 °C for 30s.

### Statistical method

Student’s *T* test was used to determine the significance between different groups. *p* < 0.05 was considered as a significant difference between compared groups.

## Results

### BIR domain is required for XIAP-mediated EGFR protein expression and anchorage-independent growth in bladder cancer cells

**Fig. 1:**
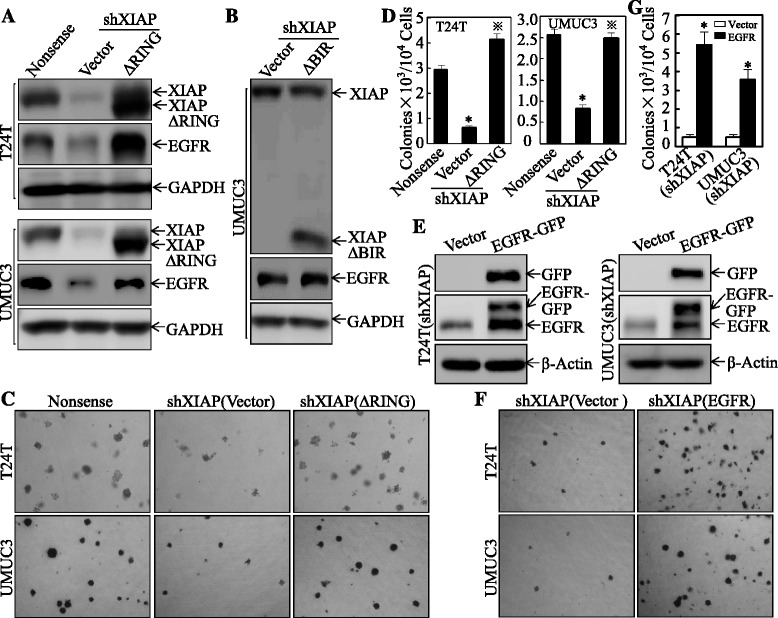
BIR domain is required for XIAP-mediated EGFR protein expression and anchorage-independent growth in bladder cancer cells. **a**, **b** The cell extracts obtained from stable transfectants, T24T(Nonsense), T24T(shXIAP/Vector), T24T(shXIAP/ΔRING), UMUC3(Nonsense), UMUC3(shXIAP/Vector), UMUC3(shXIAP/ΔRING), UMUC3(shXIAP/Vector), or UMUC3(shXIAP/ΔBIR), were subjected to Western blot for determination of expression of XIAP and EGFR. GAPDH was used as the protein loading control. **c**, **d** The indicated stable transfectants were used for determination of their anchorage-independent growth ability in soft agar assay. Colonies with more than 32 cells were scored and presented as colonies/10^4^ cells. Results were presented as means ± SD from triplicates. The *asterisk* “*” indicates a significant decrease as compared with nonsense transfectant, while symbol “※” indicates a significant increase in comparison to scramble vector transfectant (*p* < 0.05). *Error bars* represent S.D. **e** The cell extracts from T24T(shXIAP/Vector), T24T(shXIAP/EGFR-GFP), UMUC3(shXIAP/Vector), and UMUC3(shXIAP/EGFR-GFP), were subjected to Western blot for determination of indicated protein expression. β-actin was used as the protein loading control. **f**, **g** The indicated stable transfectants were used for determination of their anchorage-independent growth ability in soft agar assay. Colonies with more than 32 cells were scored and presented as colonies/10^4^ cells. Results were presented as means ± SD from three independent experiments. The *asterisk* “*” indicates a significant increase as compared with the scramble vector transfectant (*p* < 0.05). *Error bars* represent S.D.

To investigate the functional interplay between XIAP and EGFR, we first examined whether XIAP affected EGFR expression levels in human bladder cancer cells. A short-hairpin RNA (shRNA) specifically targeting human XIAP was used to knockdown endogenous XIAP in two different bladder cancer cell lines, T24T and UMUC3. The stable transfectants of control shRNA (Nonsense) and XIAP shRNA (shXIAP) were established and analyzed for EGFR expression. As shown in Fig. 1a, knockdown of XIAP in both cell lines resulted in a significant reduction of EGFR levels, suggesting a crucial role of XIAP in EGFR expression. Similar to our previous finding in XIAP−/− HCT116 cells [21], depletion of XIAP in T24T and UMUC3 cells (shXIAP) resulted in a marked reduction of anchorage-independent growth (Fig. 1c, d). Interestingly, ectopically expressing EGFR in XIAP knockdown cells completely recovered the number of colonies that grew in soft agar, indicating that reduced EGFR expression in shXIAP cells indeed contribute to the reduced anchorage-independent growth capacity of these cells (Fig. 1e–g).

XIAP contains N-terminal BIR domains and C-terminal RING domain. To identify which domain is involved in regulating EGFR expression, two XIAP mutants were generated: XIAP ∆BIR (deletion of N-terminal three BIR domains) and XIAP ∆RING (deletion of C-terminal RING domain and keeping the function of BIR domains) [18]. The stable transfectants of ∆RING and ∆BIR in XIAP knockdown cells were established (Fig. 1a, b). Interestingly, while cells overexpressing XIAP ∆RING resumed EGFR protein levels in XIAP knockdown cells, the cells overexpressing ∆BIR retained low EGFR levels similar to vector control transfectant (empty vector) (Fig. 1a, b), suggesting that BIR domain, not RING domain, plays a critical role in regulating EGFR expression. Moreover, overexpressing ∆RING not only resumed the EGFR levels but also rescued anchorage-independent growth (Fig. 1c, d). These results indicate that BIR domain is required for XIAP-dependent EGFR expression and anchorage-independent growth in human bladder cancer cells.

### XIAP BIR domain regulated the EGFR expression at translation level through increasing EGFR mRNA 3′ UTR activity

**Fig. 2:**
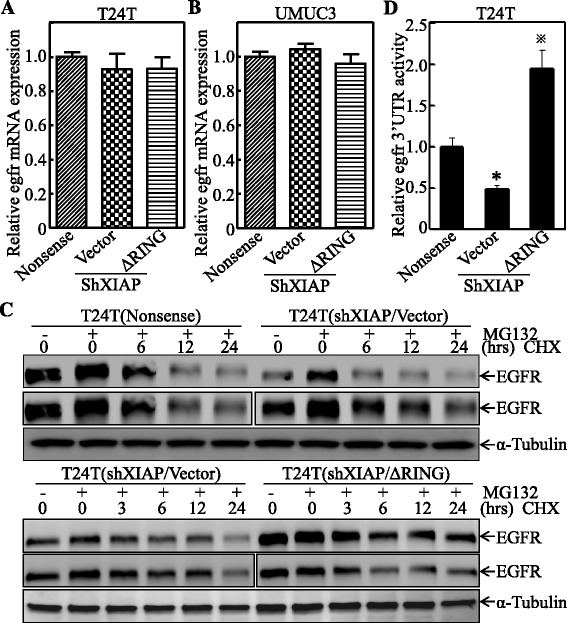
XIAP BIR domain regulated the EGFR expression at translation level through increasing EGFR mRNA 3′ UTR activity. **a**, **b** The EGFR mRNA expression level was evaluated by real-time PCR in both T24T and UMUC3 cells, and GAPDH mRNA was used as the internal loading control. Results were presented as the mean ± SD from triplicates. **c** The indicated stable transfectants were pre-treated with or without MG132 (10 μM) for 6 h and then treated with cycloheximide (CHX, 100 μg/ml) as indicated time interval. The cell extracts were subjected to Western blotting for determination of EGFR degradation, α-Tubulin was used as the protein loading control. **d** EGFR 3′ UTR luciferase reporter was stably transfected into cells as indicated, and luciferase activity was evaluated by the Dual-Luciferase Reporter Assay System. Results were presented as the mean ± SD from triplicates. The *asterisk* “*” indicates a significant inhibition as compared with nonsense transfectant, while symbol “※” indicates a significant increase in comparison to vector transfectant (*p* < 0.05). *Error bars* represent S.D.

Since overexpressing XIAP ∆BIR did not affect the EGFR expression, only XIAP ∆RING mutant was used to elucidate the molecular mechanisms underlying the XIAP regulation of EGFR expression. We first analyzed the mRNA level of EGFR in both T24T and UMUC3 stable transfectants expressing nonsense, shXIAP/Vector, and shXIAP/∆RING. As shown in Fig. 2a, b, no significant change of EGFR mRNA in all three transfectants, suggesting that XIAP-mediated regulation of EGFR expression was beyond mRNA level. To determine whether XIAP regulates EGFR protein via proteasome-mediated degradation, T24T cells expressing nonsense, shXIAP/Vector, and shXIAP/∆RING, were pretreated with or without MG132, a proteasome inhibitor, for 6 h as indicated to accumulate the EGFR protein. Cycloheximide (CHX) was then used at different time periods to observe EGFR degradation rates among the cell transfectants. The results showed that there was no observable difference between the three established cells (Fig. 2c), indicating that the difference of EGFR expression is not regulated by the proteasome-mediated protein degradation. Next, we examined the potential contribution of untranslated region (UTR) in the regulation of EGFR expression. As shown in Fig. 2d, EGFR 3′ UTR activity was inhibited in XIAP knockdown cells and overexpression of the ∆RING reversed this inhibition. Thus, XIAP BIR domain may regulate EGFR at translation level through increasing EGFR 3′ UTR activity.

### XIAP BIR domain-mediated inhibition of miR-200a was responsible for EGFR protein translational regulation by targeting EGFR mRNA 3′ UTR

**Fig. 3:**
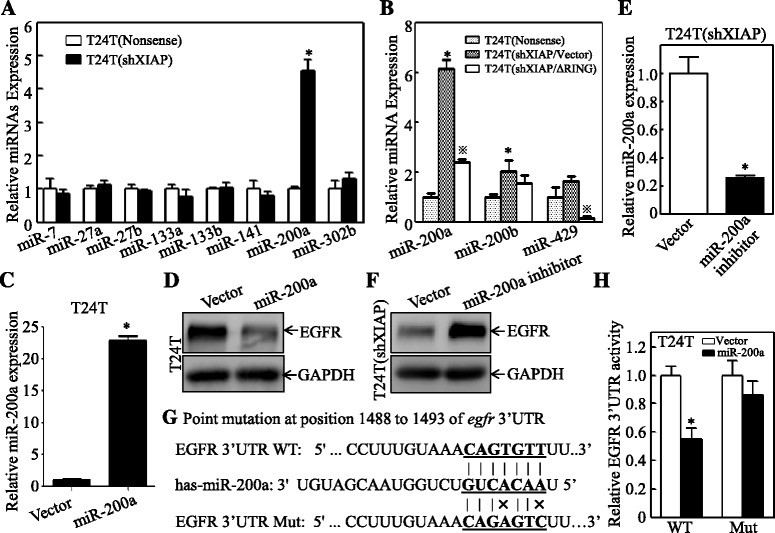
BIR domain-mediated inhibition of miR-200a was responsible for XIAP promotion of EGFR protein translational regulation by targeting EGFR mRNA 3′ UTR. **a** The indicated microRNAs, which could potential bind to EGFR mRNA 3′ UTR, were evaluated by real-time PCR. Results were presented as the mean ± SD from triplicates. The *asterisk* “*” indicates a significant increase as compared with nonsense transfectant (*p* < 0.05). **b** Expression levels of the miR-200a/200b/429 cluster in the indicated three stable cells were evaluated by real-time PCR. Results were presented as the mean ± SD of triplicates. The *asterisk* “*” indicates a significant change as compared with nonsense transfectant (*p* < 0.05), while symbol “※” indicates a significant decrease in comparison to vector transfectants (*p* < 0.05). **c** miR-200a expression plasmid was stably transfected to T24T cells and the expression efficiency was determined by real-time PCR. Results were presented as the mean ± SD of triplicates. The *asterisk* “*” indicates a significant increase as compared to that in T24T(Vector) (*p* < 0.05). **d** T24T(Vector) and T24T(miR-200a) cells were subjected to Western blotting to determine the EGFR expression. GAPDH was used as the protein loading control. **e** miR-200a inhibitor lentivirus was used to infect T24T(shXIAP) cells and the knockdown efficiency was determined by real-time PCR. Results were presented as the mean ± SD of triplicates. The *asterisk* “*” indicates a significant decrease as compared to control (*p* < 0.05). **f** The indicated cells were subjected to Western blotting to determine the EGFR expression. GAPDH was used as the protein loading control. **g** EGFR 3′-UTR Luciferase reporter and its miR-200a binding site point mutation were diagramed as indicated. **h** Wild-type (WT) and mutated (Mut) of EGFR 3′-UTR Luciferase reporters were stably co-transfected with miR-200a or its scramble vector, and the stable transfectants were used to evaluate for their reporter activity. Results were presented as the mean ± SD of triplicates. The *asterisk* “*” indicates a significant inhibition as compared with vector transfectant (*p* < 0.05). *Error bars* represent S.D.

microRNA, a class of ~22-nucletide noncoding small RNAs, had been reported to bind to the 3′ UTR of its targeted genes and inhibit their protein translation [43]. By using online prediction tools Targetscan 6.2 and starBase v2.0 [44, 45], we obtained a number of miRNAs that potentially target EGFR 3′ UTR, including miR-7, miR-27a, miR-27b, miR-133a, miR-133b, miR-141, miR-200a, and miR-302b. To determine specific miRNAs that mediate XIAP-dependent EGFR expression, we analyzed the levels of these miRNAs in T24T(Nonsense) and T24T(shXIAP) cells (Fig. 3a). The results obtained from quantitative real-time PCR indicated that only miR200a was significantly increased upon knockdown of XIAP, indicating that miR-200a is the potential miRNA that might be negatively regulated by XIAP. To further test this notion, the levels of the miR200a-200b-429 cluster were analyzed in T24T(Nonsense), T24T(shXIAP), and T24T(shXIAP/∆RING) cells. As shown in Fig. 3b, the upregulation of miR-200a by knockdown of XIAP was impaired by overexpression of BIR domain, while alteration of miR-200b and miR-429 was not observed by either knockdown of XIAP or overexpression of BIR domain, revealing the important role of the BIR domain in XIAP inhibition of miR-200a expression. To evaluate the effect of miR-200a on EGFR, we generated stable T24T transfectant expressing miR-200a, which showed over 22-fold expression of miR-200a in comparison to the corresponding empty vector transfectant (Fig. 3c). Ectopic expression of miR-200a in T24T cells led to a dramatical reduction of EGFR expression (Fig. 3d), and inhibited expression of miR-200a in T24T(shXIAP) cells could also significantly upregulate the EGFR expression (Fig. 3e, f). These results suggested that EGFR mRNA was likely to be targeted by miR-200a. To clarify if miR-200a could directly bind to 3′ UTR of the EGFR mRNA, we introduced point mutations of miR-200a binding site in EGFR 3′ UTR as indicated in Fig. 3g. The results indicated that overexpressed miR-200a failed to inhibit the mutant EGFR 3′ UTR activity, whereas it significantly inhibited the activity in wild-type EGFR 3′ UTR reporter (Fig. 3h). These results demonstrate that the BIR domain of XIAP promotes EGFR protein expression through suppression of miR-200a expression.

### XIAP BIR domain promoted miR-200a transcription by inhibiting c-Jun protein phosphorylation at Ser63/73

**Fig. 4:**
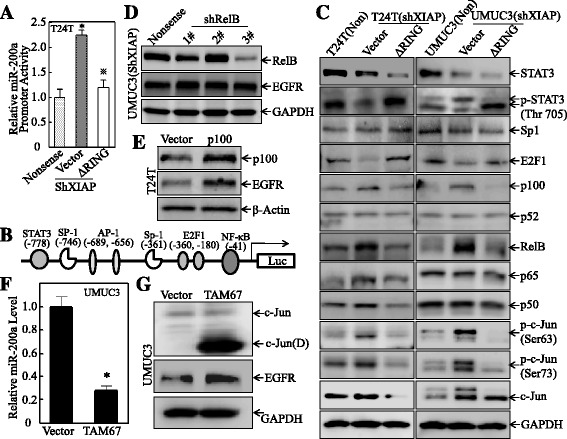
XIAP BIR domain promoted miR-200a transcription by inhibiting c-Jun protein phosphorylation at Ser63/73. **a** miR-200a promoter luciferase activity was evaluated by the Dual-Luciferase Reporter Assay System. Results are the mean ± SD of triplicates. The *asterisk* “*” indicates a significant increase as compared with nonsense cells (*p* < 0.05). The symbol “※” indicates a significant inhibition as compared with vector transfectant (*p* < 0.05). **b** The diagram of predicted transcription factor binding sites in miR-200a promoter region. **c** Western blotting was used to analyze the transcription factors expression, and GAPDH was used as the protein loading control. **d** Short hairpin RNA-specific targeting human RelB were stably transfected to UMUC3(shXIAP) cells, and Western blotting was used to determine the knockdown efficiency and EGFR expression, while GAPDH was used as the protein loading control. **e** p100 was transiently transfected to T24T cells, and Western blotting was used to determine the expression of p100 and EGFR, and β-actin was used as the protein loading control. **f** TAM67 was stably transfected into UMUC3 cells, and real-time PCR was used to determine the miR-200a expression. Results were presented as the mean ± SD of triplicates. The *asterisk* “*” indicates a significant inhibition as compared with vector transfectants (*p* < 0.05). **g** The cell extracts from UMUC3(Vector) and UMUC3(TAM67) were subjected to Western blot for determination of the indicated protein expression, and GAPDH was used as the protein loading control

To elucidate the molecular mechanisms underlying XIAP regulation of miR-200a, the promoter activity of miR-200a was evaluated and compared among T24T(Nonsense), T24T(shXIAP/Vector), and T24T(shXIAP/∆RING) cells. As shown in Fig. 4a, knockdown of XIAP significantly increased the promoter activity of miR-200a, overexpressing ∆RING in XIAP knockdown cells reversed the miR-200a promoter activity in the T24T(shXIAP/∆RING) cells. We next performed a bioinformatics scan on the promoter region of miR-200a, and several potential binding sites for transcription factors were shown in the miR-200a promoter region, including the binding sites for c-Jun, E2F1, STAT3, NF-κB, and Sp-1 (Fig. 4b). To define the specific transcription factors involved in the regulation of miR-200a, we determined the expression of these transcription factors in various stable transfectants of T24T and UMUC3 cell lines as indicated in Fig. 4c. Among these transcription factors tested, the levels of RelB, p100 protein, and c-Jun phosphorylation (Ser63/73) were upregulated in XIAP knockdown cells, and this upregulation was reversed by ectopic expression of ∆RING, suggesting they are consistent with alteration of miR-200a in those transfectants (Fig. 4c). Therefore, we knocked down RelB in UMUC3(shXIAP) cells and its effect on EGFR expression was evaluated. The results showed that knockdown of RelB exhibited little effect on EGFR expression (Fig. 4d). As a precursor of p52, p100 could function in p52-independent fashion [37]. Thus, we next evaluated the potential contribution of p100 to EGFR expression by ectopic expressing p100 in T24T cells. As shown in Fig. 4e, overexpression of p100 resulted in an upregulation of EGFR, which was inconsistent with the regulatory effect of XIAP on EGFR, excluding p100 participating in XIAP regulation of EGFR expression. Further, c-Jun dominant-negative mutant expression plasmid TAM67 was transfected into UMUC3 cells for determining the potential contribution of c-Jun activation to the expression of miR-200a and EGFR. As expected, ectopic expression of dominant-negative c-Jun (protein product named as c-Jun(D)), successfully blocked miR-200a expression (Fig. 4f) and increased EGFR protein levels (Fig. 4g), demonstrating an important role of c-Jun activation in XIAP suppression of miR-200a and upregulation of EGFR expression. Taken together, our results demonstrate that BIR domain mediates XIAP inhibition of c-Jun activation (Ser63/73 phosphorylation) and subsequently suppresses miR-200a expression and promotes EGFR protein translation.

### PP2A/MAPKK/MAPK axis was a BIR domain downstream effector mediated inhibition of c-Jun protein phosphorylation

**Fig. 5:**
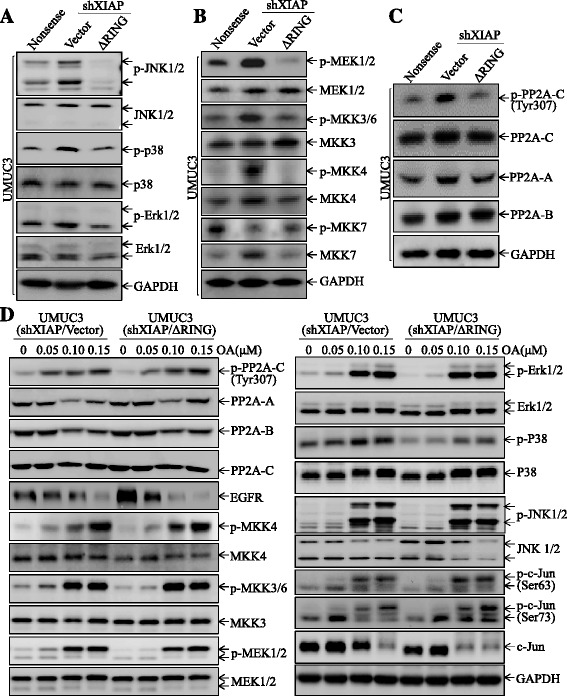
PP2A/MAPKK/MAPK axis was a BIR domain downstream effector responsible for its inhibition of c-Jun protein phosphorylation and activation. **a**–**c** Whole cells lysis obtained from the indicated transfectants were subjected to Western blot for analysis of activation of the MAPKs (**a**) and MAPKKs (**b**) and expression of PP2A. GAPDH was used as the protein loading control. **d** The indicated cells were treated with Okadaic acid (OA) for 6 h, and whole cells lyses were subjected to Western blot for determination of protein expression. GAPDH was used as the protein loading control

Phosphorylation of c-Jun at Ser63/73 is regulated by all three MAP kinases, including JNK1/2, p38, and Erk1/2. To determine which mitogen-activated protein kinase (MAPK) has involved in XIAP-mediated c-Jun inactivation, we analyzed the levels and activation of these MAPKs in UMUC3(Nonsense), UMUC3(shXIAP/vector), and UMUC3(shXIAP/∆RING). Surprisingly, all three MAPKs were activated in XIAP knockdown cells (Fig. 5a), while overexpression of ΔRING impaired those activations (Fig. 5a). Those results revealed a crucial role of BIR domain in XIAP inhibition of MAPK pathways, which led us to further examine the MAPK upstream kinases and phosphatases. As shown in Fig. 5b, almost all MAPK kinase except MKK7 exhibited the similar increases in the phosphorylation, suggesting that, instead of targeting each individual MAPK kinase (MAPKK)/MAPK, XIAP and its BIR domain might elicit a much broader effect across all MAPKK/MAPK pathways. Our previous studies have demonstrated that protein phosphatase 2 (PP2A), a major serine-threonine phosphatase that counteracts with activation of MAPKK/MAPK pathway [46], is able to regulate the c-Jun phosphorylation [18]. To test if PP2A is involved in this XIAP-regulated MAPK/c-Jun activation, we evaluated various units of PP2A protein levels in UMUC3(Nonsense), UMUC3(shXIAP/Vector), and UMUC3(shXIAP/∆RING) cells. As shown in Fig. 5c, phosphorylation of PP2A-C subunit at Tyr307 (inactivate form of PP2A) was remarkably increased in UMUC3(shXIAP/Vector) cells, but returned to the similar level observed in UMUC3(Nonsense) cells when overexpressing ΔRING domain, while other subunits, including PP2A-A and PP2A-B, did not show the consistent alteration. These results suggest that XIAP and its BIR domain provide an inhibitory effect on PP2A-C subunit phosphorylation at Tyr307 in bladder cancer cells. These results indicate that alteration of PP2A phosphorylation at Tyr307 may involve in XIAP/BIR regulation of the MAPK/c-Jun phosphorylation.

To test whether inactivation of PP2A in UMUC3(shXIAP) cells mediates activation of MAPKKs/MAPKs/c-Jun, in turn reducing EGFR expression, we treated UMUC3(shXIAP/Vector) and UMUC3(shXIAP/∆RING) cells with Okadaic acid (OA), which is a specific PP2A and protein phosphatase 1 (PP1) inhibitor [47], and the activation of MAPKKs/MAPKs/c-Jun, as well as EGFR expression, were evaluated. As shown in Fig. 5d, the treatment of cells with OA led to similar elevations of PP2A-C phosphorylation at Tyr307 accompanied by enhanced activation of MAPKKs/MAPKs/c-Jun as well as decreased EGFR expression in both UMUC3(shXIAP/Vector) and UMUC3(shXIAP/ΔRING) cells. These results indicate that the inactivation of PP2A-C by elevation of phosphorylation at Tyr307 mediates activation of MAPK/MAPKKs/c-Jun, induction of miR-200a, as well as inhibition of EGFR expression in UMUC3(shXIAP/Vector) cells, further supporting that XIAP/BIR-regulated PP2A plays an important role in their mediating EGFR expression.

### XIAP BIR domain inhibited Rac1 expression and subsequently resulted in the downregulation of PP2A-C Phosphorylation at Tyr307

**Fig. 6:**
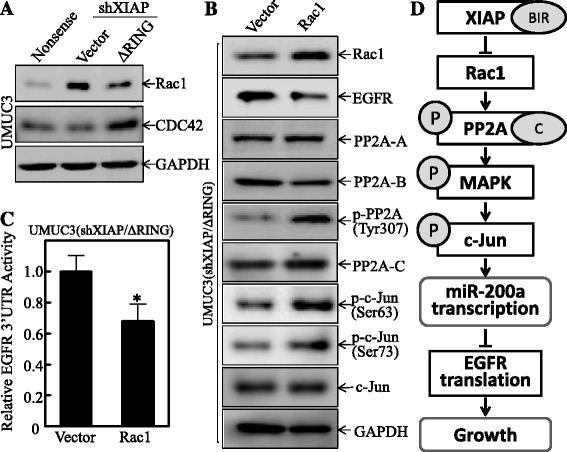
XIAP BIR domain inhibited Rac1 expression and subsequently resulted in the downregulation of PP2A-C Phosphorylation at Tyr307. **a**, **b** The whole extracts were subjected to Western blot for determination of protein expression as the indicated. GAPDH was used as the protein loading control. **c** EGFR mRNA 3′ UTR-luciferase reporter was stably transfected to UMUC3(shXIAP/ΔRING), and the luciferase activity was evaluated by the Dual-Luciferase Reporter Assay System. Results were presented as the mean ± SD of triplicates. The *asterisk* “*” indicates a significant inhibition as compared with scramble vector transfectant (*p* < 0.05). **d** The schematic diagram for the mechanisms underlying XIAP BIR promotion of EGFR protein translation and anchorage-independent growth in bladder cancer cell

The previous study identified that Rac1 could binding to SET, also known as TAF1β (template activating factor 1β), and suppress the function of PP2A by increasing the phosphorylation level in tumor cells [48, 49]. Also, it has been reported that in cardiac myocytes, Rac1/CDC42 complex promotes PP2A activity by dephosphorylating PP2A through upregulating p21-activated kinase-1 (Pak1) activity [50]. To determine whether Rac1 and CDC42 were involved in the BIR regulation of PP2A, we evaluated the expression of Rac1 and CDC42 in UMUC3(Nonsense), UMUC3(shXIAP/Vector), and UMUC3(shXIAP/ΔRING) cells. The results showed that knockdown of XIAP remarkably increased the Rac1 expression and the introduction of ∆RING reversed the Rac1 expression (Fig. 6a). In contrast to Rac1, CDC42 showed the opposite alterations (Fig. 6a), which minimized the possibility of Rac1/CDC42 complex participating in XIAP regulation of PP2A activity and further suggesting that Rac1 might contribute to this regulation. As expectedly, overexpression of Rac1 in UMUC3(shXIAP/ΔRING) cells led to downregulation of EGFR and upregulation of the PP2A-C phosphorylation at Tyr307 as well as c-Jun phosphorylation at Ser63/Ser73 (Fig. 6b). Consistently, the 3′ UTR activity of EGFR was significantly downregulated in the same cells (Fig. 6c). These data demonstrate that XIAP and its BIR domain inhibit Rac1 expression and subsequently decreasing the PP2A-C phosphorylation at Tyr307 and MAPKK/MAPK/c-Jun activation, and further resulting in miR-200a induction and EGFR translation inhibition, as diagramed in Fig. 6d.

## Discussion

Although recent studies show the co-overexpression of XIAP and EGFR in many cancers [14, 35, 51, 52], and the high sensitive to anti-IAPs therapy if the cancers with EGFR overexpression [35], a possible association of XIAP with EGFR overexpression has never been explored in previous studies. We demonstrate here that XIAP is a strong positive regulator of EGFR expression in human bladder cancers and that XIAP BIR domain plays an important role in the mediation of EGFR protein expression by promoting its protein translation. We find that BIR domain of XIAP inhibits the Rac1 protein expression, which leads to the decrease of PP2A phosphorylation at Tyr307, in turn resulting in the activation of the PP2A activity and consequently inactivating MAPKK/MAPK/c-Jun axis. The inhibition of c-Jun further leads to a reduction of miR-200a transcription. Since miR-200a binds to the 3′-UTR of EGFR mRNA and inhibits EGFR protein translation, the attenuation of miR-200a expression by BIR domain of XIAP results in the enhancement of EGFR protein translation and increases the anchorage-independent growth of the bladder cancer cells. This is the first demonstration that XIAP promotes the anchorage-independent growth of human bladder cancer cell via positive regulation of EGFR translation.

There is increasing evidence showing that XIAP functions much more than its ability to inhibit apoptosis. Our recent studies have revealed several non-apoptosis-related functions of XIAP via its RING domain, including the upregulation of cyclin D1, while promoting bladder cancer cell growth [18], and the promotion of F-actin formation and colon cancer cell invasion via inhibition of SUMOlation of RhoGDIα (Rho GDP-dissociation inhibitor α) at lys-138 [53]. Our most recent studies also reveal that the RING domain of XIAP promotes Sp1-mediated transcription of miR-4295, which targets the 3′ UTR of p63α mRNA, for inhibiting p63α translation and enhancing urothelial transformation [54]. In the dissection of BIR domain functions, we find that BIR domains can directly bind to E2F1 (E2F transcription factor 1) and increase its transactivation and cyclin E expression [21]. In the current studies, we identify a novel function of BIR domains in the promotion of bladder cancer cell growth by upregulation of EGFR expression. We find that knockdown XIAP attenuated EGFR expression and the anchorage-independent growth ability, while ectopic expression of XIAP BIR domains in the XIAP knockdown cells restores the EGFR level and the anchorage-independent growth ability. The deficiency of EGFR expression and anchorage-independent growth ability in the XIAP knockdown cells is also reversed by ectopic expression of EGFR. This is the first demonstration of XIAP BIR domains as a potent positive regulator of EGFR expression, which in turn promotes bladder cancer cell anchorage-independent growth.

Nearly a quarter, between 20 and 30%, of human bladder cancers are muscle-invasive bladder cancers, and half of the patients diagnosed with muscle-invasive bladder cancer will die from this aggressive disease within 5 years [55, 56]. Thus, effective targeted therapeutic agents are urgently needed. Due to their multidimensional roles in the progression of cancers, XIAP and its family members have emerged as attractive candidates for anti-cancer therapy [57, 58]. A study shows that high level of XIAP expression correlates with tumor differentiation and significantly lower recurrence-free survival rates and independently predicting the recurrence of non-muscular invasive bladder cancer in a multivariate analysis [59]. In addition to XIAP, EGFR is also a well-known tumor therapeutic target. EGFR is overexpressed in basal-like muscle-invasive bladder cancers, which is sensitive to anti-EGFR therapy [25]. Other studies have shown that EGFR overexpressed tumors are more sensitive to anti-IAPs therapy [35] and XIAP also can lead to the resistance to anti-EGFR therapy [60]. Our studies here found that the overexpression of EGFR was attributed to BIR domain of abnormally expressed XIAP in bladder cancers (BCs), which offers an exciting new opportunity for us to explore the potential usage of XIAP BIR domain as a therapeutic target for invasive BCs, which may avoid the issue of XIAP related anti-EGFR therapy resistance, therefore in turn helping to improve the clinical outcome of patients with invasive BCs.

In this study, we also show a new mechanism for XIAP BIR domain in regulating miR-200a through Rac1/PP2A/MAPKK/MAPK/c-Jun axis. miR-200a, the anti-tumor microRNA, is able to inhibit the epithelial-mesenchymal transition and reverse the resistance to anti-EGFR therapy [61, 62]. Our results showed that miR-200a could bind to EGFR 3′-UTR and inhibit EGFR translation. Rac1, a well-known Rho GTPase, plays important roles in numerous cellular functions [63]. It had been demonstrated that XIAP BIR domains are direct E3 ubiquitin ligases of Rac1 [64]. Also, Rac1 has been reported to form a complex with SET and decrease the PP2A activity through increased PP2A phosphorylation level [48]. We found here that downregulation of XIAP resulted in an upregulated Rac1 and higher phosphorylation of PP2A, while ectopic overexpression of Rac1 could also lead to a higher phosphorylation level of PP2A. Further analysis revealed that the knockdown of XIAP could lead to a high phosphorylation level of PP2A, and subsequently activate the MAPK kinase/MAPK pathway, leading to a high phosphorylation level of c-Jun at ser63 and ser73, which could bind to the miR-200a promoter region and promote the transcription of miR-200a. Upon the treatment of cells with OA, a PP2A specific inhibitor, the phosphorylation of MAPK and MAPK kinases were markedly upregulated, while EGFR expression was dramatically inhibited. Taken together, we therefore demonstrate that BIR domains regulated the miR-200a transcription through the PP2A-related MAPK/c-Jun activation for the first time. It was noted that an increased activation of MAPKs was associated with a reduction of EGFR expression and tumor growth. A similar observation has also been reported in previous studies showing that the inhibited ERK activity promotes the EGFR activation and augments EGFR-driven motility of prostate cancer cells [65]. Although detailed mechanisms underlying this observation are not explored yet, we anticipate that the comprehensive feedback regulatory pathways might be involved. Further elucidation of this issue will be helpful for understanding the nature of XIAP in the regulation of BC growth.

## Conclusions

In conclusion, we demonstrate a novel function of XIAP BIR domain in regulating cancer cell anchorage-independent growth ability through the upregulation of EGFR translation via the inhibition of miR-200a transcription through the Rac1/PP2A/MAPKK/MAPK/c-Jun axis. This function provides new insight into the mechanisms behind the XIAP regulating the cancer cell growth. Altogether, the current studies provide very useful information for new anti-XIAP therapeutic drug designs and should in turn help to improve clinical outcomes of BC patients with XIAP/EGFR overexpression.

## Abbreviations

BC: Bladder cancer
BIR: Baculovirus IAP repeat domains
CHX: Cycloheximide
c-Jun(D): Dominant-negative c-Jun
EGFR: Epidermal growth factor receptor
IAP: Inhibitors of apoptosis
MAPK: Mitogen-activated protein kinase
MAPKK: MAPK kinase
NSCLC: Non-small-cell lung cancer
OA: Okadaic acid
Pak1: p21-activated kinase-1
PP1: Protein phosphatase 1
PP2A: Protein phosphatase 2
shRNA: Short hairpin RNA
TAF1β: Template activating Factor 1β
TGFα: Transforming growth factor α
UTR: Untranslated region
XIAP: X-linked inhibitor of apoptosis protein
ΔBIR: Deletion of N-terminal three BIR domains
ΔRING: Deletion of C-terminal RING domain
